# IP_3_ Receptor-Mediated Calcium Signaling and Its Role in Autophagy in Cancer

**DOI:** 10.3389/fonc.2017.00140

**Published:** 2017-07-05

**Authors:** Elzbieta Kania, Gemma Roest, Tim Vervliet, Jan B. Parys, Geert Bultynck

**Affiliations:** ^1^Laboratory for Molecular and Cellular Signaling, Department of Cellular and Molecular Medicine, Leuven Kankerinstituut, KU Leuven, Leuven, Belgium

**Keywords:** Ca^2+^ signaling, inositol 1,4,5-trisphosphate, inositol 1,4,5-trisphosphate receptors, autophagy, apoptosis, cancer

## Abstract

Calcium ions (Ca^2+^) play a complex role in orchestrating diverse cellular processes, including cell death and survival. To trigger signaling cascades, intracellular Ca^2+^ is shuffled between the cytoplasm and the major Ca^2+^ stores, the endoplasmic reticulum (ER), the mitochondria, and the lysosomes. A key role in the control of Ca^2+^ signals is attributed to the inositol 1,4,5-trisphosphate (IP_3_) receptors (IP_3_Rs), the main Ca^2+^-release channels in the ER. IP_3_Rs can transfer Ca^2+^ to the mitochondria, thereby not only stimulating core metabolic pathways but also increasing apoptosis sensitivity and inhibiting basal autophagy. On the other hand, IP_3_-induced Ca^2+^ release enhances autophagy flux by providing cytosolic Ca^2+^ required to execute autophagy upon various cellular stresses, including nutrient starvation, chemical mechanistic target of rapamycin inhibition, or drug treatment. Similarly, IP_3_Rs are able to amplify Ca^2+^ signals from the lysosomes and, therefore, impact autophagic flux in response to lysosomal channels activation. Furthermore, indirect modulation of Ca^2+^ release through IP_3_Rs may also be achieved by controlling the sarco/endoplasmic reticulum Ca^2+^ ATPases Ca^2+^ pumps of the ER. Considering the complex role of autophagy in cancer development and progression as well as in response to anticancer therapies, it becomes clear that it is important to fully understand the role of the IP_3_R and its cellular context in this disease. In cancer cells addicted to ER–mitochondrial Ca^2+^ fueling, IP_3_R inhibition leads to cancer cell death *via* mechanisms involving enhanced autophagy or mitotic catastrophe. Moreover, IP_3_Rs are the targets of several oncogenes and tumor suppressors and the functional loss of these genes, as occurring in many cancer types, can result in modified Ca^2+^ transport to the mitochondria and in modulation of the level of autophagic flux. Similarly, IP_3_R-mediated upregulation of autophagy can protect some cancer cells against natural killer cells-induced killing. The involvement of IP_3_Rs in the regulation of both autophagy and apoptosis, therefore, directly impact cancer cell biology and contribute to the molecular basis of tumor pathology.

## Intracellular Ca^2+^ Signaling: The Endoplasmic Reticulum (ER), Mitochondria, and Lysosomes

Intracellular Ca^2+^ signaling controls a plethora of cellular processes, including secretion, gene transcription, metabolism, and cell death, thereby impacting cell function and cell survival (1–3). Intracellular Ca^2+^ signals are characterized by their spatiotemporal properties. As a function of time, Ca^2+^ signals can occur as transient increases in [Ca^2+^] (Ca^2+^ oscillations) (4) or as more sustained increases in [Ca^2+^] (global Ca^2+^ transients) (5). As a function of space, Ca^2+^ signals can occur in localized domains near the plasma membrane (PM) or organelles, such as the ER, mitochondria, lysosomes, Golgi, and nucleus (3, 6). Localized Ca^2+^ signaling is established in so-called microdomains due to the close apposition of different organellar compartments (7–9) or of organelles with the PM through molecular tethers (10–12).

The major intracellular Ca^2+^-storage organelle is the ER, where most of the intracellular Ca^2+^ is accumulated *via* active Ca^2+^ transport mediated by sarco/endoplasmic reticulum Ca^2+^ ATPases (SERCA) followed by intraluminal Ca^2+^ buffering by calreticulin, calnexin, and other Ca^2+^-binding proteins (13, 14). These mechanisms allow for an adequate Ca^2+^ filling of the ER, which is required for the activity of molecular chaperones and the folding of enzymes. Hence, a depletion of the ER Ca^2+^ stores leads to ER stress, a condition associated with impaired protein folding capacity (13, 15, 16). To cope with this, cells engage the unfolded protein response, a concerted program triggered through the three classical ER stress sensors: inositol-requiring enzyme1α, RNA-dependent protein kinase-like ER kinase, and activating transcription factor 6 (17). Mild or transient ER stress induces activity of chaperones, folding enzymes, reactive oxygen species (ROS) scavengers, and degradative pathways, such as autophagy, while severe or persistent ER stress induces cell death (18–20).

These Ca^2+^-uptake mechanisms are counteracted by, on the one hand, Ca^2+^-leak channels and, on the other hand, Ca^2+^-release channels (21). Ca^2+^-leak channels establish a constitutive, passive Ca^2+^ leak from the ER, preventing ER Ca^2+^ overload that would result in cell death. Different ER Ca^2+^-leak channels have been identified, likely all contributing to this passive Ca^2+^ leak to some extent, although it is possible that some of these channels are restricted to certain cell types or systems (22–25). Presenilin 2, Ca^2+^ release-activated Ca^2+^ channel protein 2 (Orai2) and inositol 1,4,5-trisphosphate (IP_3_) receptor (IP_3_R) isoform 1 (IP_3_R1) have been proposed as major ER Ca^2+^-leak channels, based on a systems biology approach using HeLa cells (26). However, the ER Ca^2+^-leak rates in wild-type HEK293 versus HEK293 cells lacking all three IP_3_R isoforms, directly measured by using a genetically encoded ER Ca^2+^ sensor, were very similar, indicating that at least in HEK cells and these experimental conditions, IP_3_R does not contribute in an important way to the passive Ca^2+^ leak from the ER (27). These Ca^2+^-leak channels impact the steady-state Ca^2+^ content of the ER, which determines the Ca^2+^ available for release upon agonist stimulation (28, 29). Ca^2+^ release from the ER occurs through IP_3_Rs (30, 31) or ryanodine receptors (RyRs) (32–34). IP_3_Rs are ubiquitously expressed and are activated in response to IP_3_, which is produced from phosphatidylinositol 4,5-bisphosphate (PIP_2_) upon hydrolysis mediated by phospholipase C (35). This typically happens in response to cellular stimulation with hormones, growth factors, neurotransmitters, or antibodies. However, it is clear that many cells even in basal, non-stimulated conditions, display a constitutively low level of IP_3_-mediated signaling. The Ca^2+^ depletion of the ER, resulting from Ca^2+^ release, can trigger the activation of store-operated Ca^2+^ entry (SOCE) through stromal interaction molecule 1 (Stim1)-dependent activation of Ca^2+^ release-activated Ca^2+^ channel protein 1 (Orai1) channels (36–38).

Ca^2+^ release from the ER does not only result in a [Ca^2+^] rise in the cytosol but also leads to [Ca^2+^] increase in other organelles, including the mitochondria and the lysosomes (6, 39). This is due to contact sites between ER and mitochondria and between ER and lysosomes, decreasing the distance between these organelles and the ER (39–43). In addition to this, a highly negative potential of about −180 mV exists across the mitochondrial inner membrane, establishing a strong electrochemical driving force for Ca^2+^ uptake in the mitochondria (40). It is well known that IP_3_R-mediated Ca^2+^ release from the ER can “quasi-synaptically” transfer into the mitochondria (7, 44). This occurs *via* the so-called mitochondria-associated ER membranes (MAMs) that also harbor the IP_3_R and the voltage-dependent anion channel type 1 that permeates Ca^2+^ across the mitochondrial outer membrane (39, 41, 42). Once in the mitochondrial intermembrane space, Ca^2+^ is transported across the mitochondrial inner membrane *via* the mitochondrial Ca^2+^ uniporter (MCU) (45). The MCU has low inherent affinity for Ca^2+^ and is highly cooperative due to accessory proteins such as MICU1 (46). By comparison, Ca^2+^ uptake into the lysosomes is much less understood. Nevertheless, it is anticipated that a strong electrochemical gradient for H^+^ is present resulting from a low lysosomal pH (~4–5) which can be used for lysosomal Ca^2+^ accumulation *via* the lysosomal H^+^/Ca^2+^ exchanger (47, 48). Ca^2+^ can be released from these lysosomal Ca^2+^ stores *via* a variety of channels, including two-pore channels 1/2 (TPC1/2) and transient receptor potential superfamily channels such as TRPML1 (mucolipin1/MCOLN1)(47, 49–52). An important second messenger triggering lysosomal Ca^2+^ release through TPC1/2 is nicotinic acid adenine dinucleotide phosphate (NAADP) (53–55). TRPML1 present in endolysomal vesicles can be activated by phospholipids such as phosphatidylinositol 3,5-bisphosphate [PI(3,5)P_2_] (56, 57).

## Ca^2+^ Signaling in Autophagy

### ER-Derived Ca^2+^ Signaling in Autophagy

**Figure 1:**
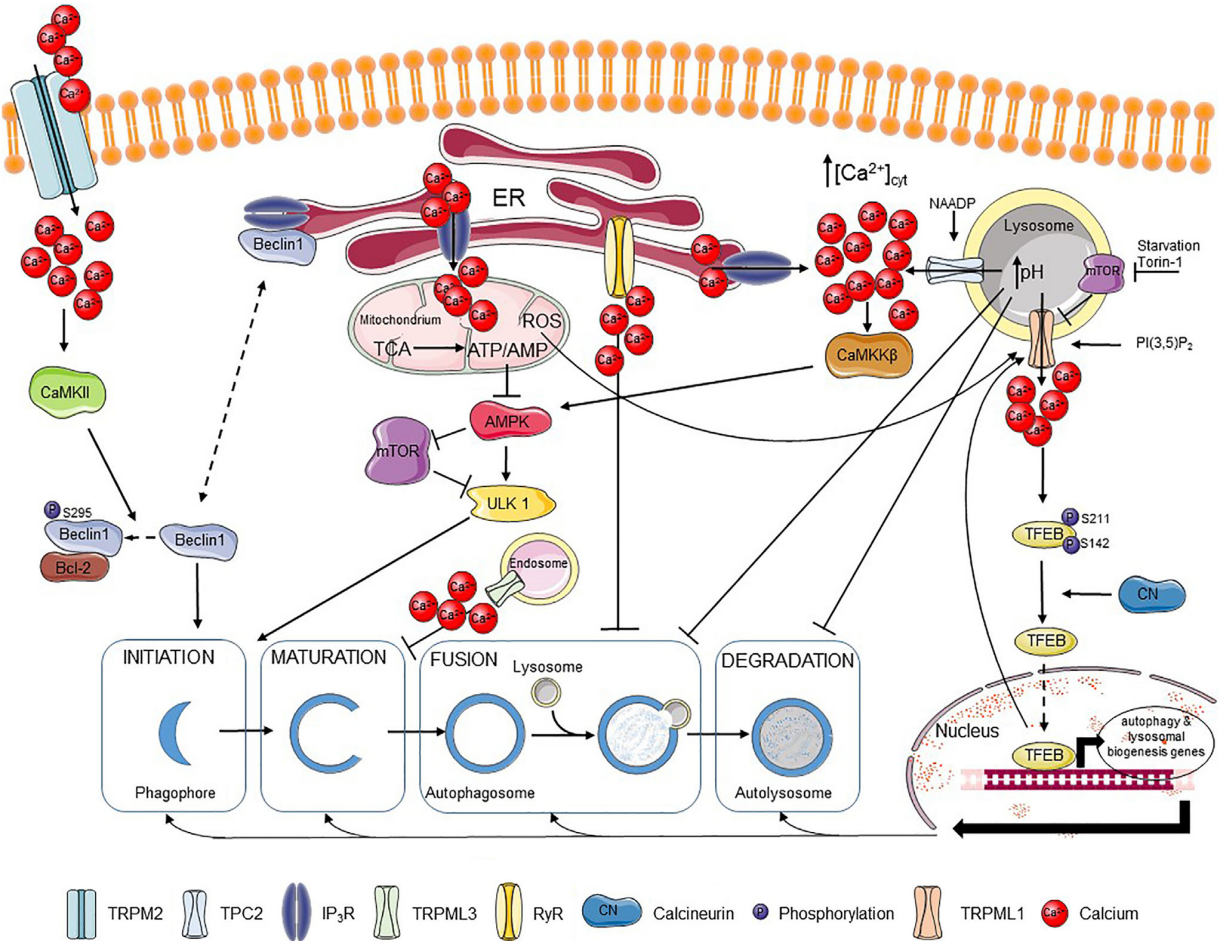
Ca^2+^ regulates different steps of autophagy. Extracellular Ca^2+^ can enter the cell *via* the TRPM2 channel, where it activates CaMKII that phosphorylates Beclin 1, promoting its binding to Bcl-2 and blocking autophagy initiation. Beclin 1 can also be scaffolded to the IP_3_R, which also limits autophagy promotion. IP_3_Rs are responsible for fueling mitochondria with Ca^2+^, which supports the tricarboxylic acid (TCA) cycle and the resulting ATP production. The presence of an adequate ATP/AMP ratio suppresses the AMP-activated kinase (AMPK)–mechanistic target of rapamycin (mTOR)–unc-51-like kinase 1 (ULK1) axis and thus autophagy induction. Autophagosome maturation can be inhibited by the TRPML3 channel mostly expressed in endosomes, while fusion of autophagosomes and lysosomes can be blocked by basal RyR activity. Ca^2+^ release through lysosomal nicotinic acid adenine dinucleotide phosphate (NAADP)-activated TPC2 channels can be further amplified by Ca^2+^ release from IP_3_Rs resulting in stimulation of the CaMKKβ–AMPK pathway and autophagosome formation. Ca^2+^ release from the lysosomes can, however, affect lysosomal pH and so abrogate fusion of lysosomes with autophagosomes as well as the further lysosome-dependent degradation. TRPML1, another lysosomal channel, can be regulated by different factors, including lysosomal mTOR, PI(3,5)P_2_ or reactive oxygen species (ROS). Ca^2+^ released from TRPML1 activates calcineurin, which binds and dephosphorylates TFEB and promotes its nuclear translocation, where TFEB induces transcription of various autophagy-related and lysosomal biogenesis genes. Plain black arrows indicate activatory and inhibitory pathways; dashed arrows indicate intracellular movement.

It is well-established that Ca^2+^ signaling impacts autophagy initiation and progression (Figure 1). Ca^2+^ signaling modulates as well basal autophagic flux as mechanistic target of rapamycin (mTOR)-controlled autophagic flux, induced by nutrient starvation or rapamycin (58–60). Moreover, compounds that directly affect Ca^2+^ signaling, including agonists, Ca^2+^ ionophores, and SERCA inhibitors that indirectly cause ER Ca^2+^-store depletion *via* the basal Ca^2+^ leak, can modulate the autophagic process (61). The ER, as the main intracellular Ca^2+^-storage organelle, has been implicated in controlling basal autophagy. This is related to Ca^2+^-dependent energizing of the mitochondria (62). Basal and constitutive Ca^2+^-release events from the ER, mediated by IP_3_R channels, have been involved in sustaining mitochondrial bioenergetics by driving NADH production and subsequent ATP synthesis by continuously providing Ca^2+^ to the mitochondria (62) (Figure 1). This is due to the presence of three mitochondrial Ca^2+^-dependent tricarboxylic acid (TCA) cycle enzymes, which activities are enhanced by mitochondrial Ca^2+^ (40). Abrogating these Ca^2+^ signals through pharmacological inhibition or genetic knock down of IP_3_Rs resulted in an increased autophagic flux due to an increase in the activity of the AMP-activated kinase (AMPK), a positive regulator of autophagy (i) inhibiting mTOR and (ii) activating the unc-51-like kinase 1 (ULK1) complex (63). However, the increased basal autophagic flux triggered upon IP_3_R inhibition appeared independent on mTOR (62) (Figure 1). Thus, IP_3_Rs exert an inhibitory role on basal autophagy, and consequently IP_3_R inhibition results in increased basal autophagy. An additional mechanism for this autophagy-inhibitory role of IP_3_Rs has been attributed to its Beclin 1-scaffolding function (64, 65). Beclin 1 regulates autophagy by forming a complex with the class III phosphatidylinositide 3-kinase Vps34, thereby stimulating phagophore nucleation, an early step in the autophagy process (66–68). It has been shown that the IP_3_R, independently of its Ca^2+^-flux properties, could serve as a sink for Beclin 1 recruitment, reducing the availability of free Beclin 1 to drive autophagy (69) (Figure 1).

Besides IP_3_Rs, RyRs have also been implicated in autophagy. In hippocampal neuronal stem cells, insulin withdrawal resulted in the upregulation of the RyR3 isoform, which triggered cell death through increased autophagy. Activation of RyRs using caffeine increased autophagic cell death, while their inhibition using dantrolene suppressed this process (70). However, more recently, RyRs, both endogenously expressed in skeletal muscle cell lines and in dissociated rat hippocampal neurons or ectopically expressed in HEK cells, have been implicated in inhibition of autophagic flux, particularly at the level of autophagosome/lysosome fusion (Figure 1). RyR inhibition resulted in an increase of autophagic flux, independent of mTOR activity or of early autophagy regulators (71). This indicates that both IP_3_Rs and RyRs could suppress basal autophagy, but by acting at a different level: IP_3_Rs by suppressing autophagy at a proximal level by driving mitochondrial bioenergetics and thus decreasing AMPK activity, while RyRs block autophagy at a distal level by counteracting the fusion of autophagosomes and lysosomes.

In contrast to this, Ca^2+^ mobilization from the ER into the cytosol by itself can augment autophagic flux. Both physiological agonists as well as chemicals, such as Ca^2+^ ionophores and SERCA inhibitors, that provoke [Ca^2+^] rises in the cytosol originating from the ER result in the activation of Ca^2+^/calmodulin-dependent kinase kinase β (CaMKKβ) that is an upstream activator of AMPK and autophagy (72). In fact, mechanisms that limited cytosolic [Ca^2+^] rise induced by these agents also limited autophagy induction. As such, Bcl-2, a known negative regulator of autophagy, was proposed not only to limit autophagy by scaffolding Beclin 1 but also by reducing the ER Ca^2+^-store content, thus suppressing cytosolic [Ca^2+^] rises and the extent of CaMKKβ activation (Figure 1) (73).

In addition to this, cytosolic [Ca^2+^] rises can promote autophagosome formation by recruiting the phosphatidylinositol 3-phosphate [PI(3)P]-binding protein, encoded by autophagy-related gene (Atg) 18 (WIPI1/WIPI2), to autophagosomal membranes. Atg18, together with Atg16L and ULK1, are recruited to early autophagosomal structures and are critical for the formation of LC3-positive autophagosomes (74). Furthermore, the autophagosomal recruitment of Atg18 was blocked by cytosolic Ca^2+^ chelation. This mechanism allowed for an induction of autophagy by cytosolic [Ca^2+^] rise independently of AMPK activation or mTOR inhibition (75). However, apart from the autophagy-inducing properties of thapsigargin and Ca^2+^ ionophores, these agents have also been reported to suppress autophagosome biogenesis at steps in the autophagy pathway subsequently to WIPI1 punctae formation but preceding autophagosome closure (76). This may relate to the disturbance of critical Ca^2+^ fluxes from the ER during the distal steps in the autophagy process, including the closure of the autophagosomal vesicles.

Beyond these roles in modulating basal autophagy, the ER and more particular ER-derived Ca^2+^ signals mediated *via* IP_3_Rs have been involved in driving starvation- and rapamycin-induced autophagy (58, 59). Starvation and rapamycin are two triggers that induce autophagy through inhibition of mTOR, a negative regulator of autophagy. Starvation- and rapamycin-induced autophagy resulted in enhanced Ca^2+^ signaling from the ER through a mechanism that involved complex formation between IP_3_Rs and Beclin 1 and a subsequent direct IP_3_R sensitization by Beclin 1 (58) (Figure 1). In turn, cytosolic Ca^2+^ and IP_3_R activity were critical for cells to be able to increase their autophagic flux in response to nutrient starvation and rapamycin.

Resveratrol (RSV) is a polyphenol found in some food products and in red wine. Several health-promoting effects have been attributed to RSV, including longevity, anti-aging, and improved cardiovascular health (77, 78). These beneficial effects of RSV have been linked to its ability to induce autophagy. RSV induces autophagy in a pleiotropic manner *via* both mTOR-dependent and -independent mechanisms. RSV can activate the deacetylases sirtuins, a positive regulator of AMPK (79, 80). RSV can also directly inhibit mTOR by docking onto its ATP-binding pocket and thus competing with ATP. The inhibition of mTOR and the presence of ULK1 appeared to be critical for RSV-induced autophagy (81). However, RSV can also promote autophagy in a non-canonical manner, whereby RSV induces autophagosome formation independently of Beclin 1 or its binding partner Vps34 (82, 83). Also, Ca^2+^ signaling has been implicated in RSV-induced autophagy (27). RSV can deplete the ER Ca^2+^ stores independently of the presence or absence of IP_3_Rs (27), which in part may be due to its inhibitory effect on mitochondrial ATP production, thereby suppressing SERCA-mediated ER Ca^2+^ uptake (84). Yet, although RSV triggered a Ca^2+^ leak from the ER independently of IP_3_Rs, the ability of RSV to induce autophagy was critically dependent on the presence of IP_3_Rs and on the availability of cytosolic Ca^2+^ (27). In this study, the inhibitory effect of RSV on mTOR activity was confirmed and did neither require cytosolic Ca^2+^ nor IP_3_R expression (27).

### Endolysosomal-Derived Ca^2+^ Signaling in Autophagy

A genetic analysis of mucolipidosis type IV (MLIV), a lysosomal storage disease associated with severe neurological deficiencies, implicated that mutations in TRPML1 play a role in autophagy deregulation (85). Fibroblasts derived from MLIV patients expressing mutant TRPML1 displayed an increased autophagosome formation accompanied with a delay in the fusion of autophagosomes with lysosomes. This was proposed to contribute to an accumulation of p62 and a defective removal of ubiquitinated proteins and/or defective mitochondria (85). Also, chaperone-mediated autophagy (CMA) was defective in MLIV fibroblasts, which could be attributed to TRPML1’s ability to bind and recruit Hsc70 and Hsp40 proteins, two components critical for CMA, and a subsequent reduction in lysosomal LAMP2A-protein levels. As a consequence, oxidized proteins may accumulate in the cytosol due to their impaired degradation *via* CMA (86). However, in these studies, the role of TRPML1 channel activity and lysosomal Ca^2+^ release was not addressed.

One of the first data linking TRPML-mediated Ca^2+^ release and the autophagy pathway was provided from overexpression studies using TRPML3/MCOLN3 (87). This channel is mainly present in early endosomal compartments, where pH is not as low as in lysosomes. These endosomal compartments may host more functional TPRML3 channels than lysosomes, as low pH appears to inactivate TRPML3-mediated Ca^2+^ flux. Overexpression of TRPML3/MCOLN3 not only resulted in severe changes in the endosomal pathway, including increased endosomal pH, but also in defective autophagosome maturation (87) (Figure 1).

More recently, a direct role of lysosomal Ca^2+^ release through TRPML1 channels in upregulating autophagy upon mTOR inhibition has been elucidated (88). Nutrient starvation resulted in rapid peri-lysosomal [Ca^2+^] rises, in the close proximity of TRPML1 channels. These [Ca^2+^] rises were concentrated around lysosomes and could not be observed in the bulk cytosol. In turn, [Ca^2+^] rises resulted in the activation of the Ca^2+^/calmodulin-dependent phosphatase calcineurin, which can dephosphorylate the transcription factor TFEB. As a consequence, upon nutrient starvation, calcineurin dephosphorylated TFEB at two residues (Ser142 and Ser211) regulating TFEB nuclear translocation, resulting in nuclear accumulation of TFEB and activation of genes necessary for autophagy and lysosomal biogenesis (88) (Figure 1).

TRPML1 itself is also regulated during nutrient starvation. TRPML1 and the hereby associated Ca^2+^ flux from the lysosomes became upregulated upon nutrient deprivation, which was accompanied by transcription of autophagy-regulating genes (89). TRPML1 upregulation also occurred in response to complete inhibition of the mTOR complex 1 by Torin-1, while this was not observed upon treatment with rapamycin, a partial, allosteric inhibitor of mTORC1. A critical role for TFEB was found in the upregulation of TRPML1. Both starvation and Torin-1 treatment were able to induce TFEB dephosphorylation and its nuclear translocation, while rapamycin failed to do this. A more direct link between TFEB activation and TRPML1 upregulation was shown by overexpressing constitutively dephosphorylated and thus active TFEB in cells, which resulted in a functional upregulation of TRPML1 channels. The upregulation of TRPML1 activity by TFEB could be partially attributed to an increase in mRNA and protein expression of the channel, but likely also involved post-translational modifications or upregulation of TRPML1-interacting/modulating proteins. Moreover, a role of the lysosomal lipid PI(3,5)P_2_, a TRPML1-activating lipid which levels decrease upon nutrient starvation, was proposed as part of a compensatory mechanism that causes upregulation of TRPML1. At the functional level, TRPML1 activity was critical for the increase in lysosomal proteolytic activity induced by nutrient starvation (89).

Interestingly, TRPML1 activity is not only controlled by PI(3,5)P_2_ levels and *via* the TFEB transcription factor but also directly by mTOR (90). In nutrient-replete conditions, when mTOR was active and autophagy was suppressed, mTOR phosphorylated two serine residues in the C-terminal tail of TRPML1, resulting in TRPML1 channel inhibition. Upon mTOR inhibition by rapamycin, leading to autophagy induction, TRPML1 became dephosphorylated and active, resulting in lysosomal Ca^2+^ release. Moreover, rapamycin could induce Ca^2+^ release in cells expressing wild-type TRPML1 but not in cells expressing TRPML1 in which the two phosphorylable serine residues were mutated. Thus, loss of TRPML1 phosphorylation upon mTOR inhibition results in increased TRPML1 activity, driving autophagic flux (90). However, at this point, it is not clear which phosphatase is responsible for dephosphorylating the serine residues that are phosphorylated by the mTOR kinase.

Recently, it was shown that TRMPL1 can serve as a redox status sensor and can release Ca^2+^ upon stimulation by ROS or by mitochondrial uncouplers (91, 92). As a result of this Ca^2+^ release, also calcineurin-dependent TFEB activation and nuclear translocation occurred, which could be blocked by the ROS scavenger N-acetyl-cysteine (NAC), as well as by BAPTA-AM or synthetic TRPML1 inhibitors (ML-SIs). Since mitochondrial uncouplers failed to stimulate nuclear translocation of TFEB in TRPML1 knockout (KO) cells, this points out that TRPML1 is specifically required for ROS-induced activation of TFEB. By contrast, functional TRMPL1 was not required for TFEB nuclear translocation induced by mTOR inhibition through Torin-1 or nutrient starvation (91). In addition, ROS-induced autophagy and lysosome biogenesis could be impeded by NAC treatment as well as by ML-SIs or TRPML1 KO (91).

In contrast to TRPML1-mediated Ca^2+^ release from the lysosomes, thereby positively regulating autophagy, TPC2 has been implicated in the inhibition of autophagy (93). TPC2 channels are activated by NAADP. Overexpression of TPC2 resulted in the accumulation of autophagosomes, a phenomenon boosted by NAADP but antagonized by either the NAADP antagonist Ned19 or by knockdown (KD) of the essential autophagy gene Atg5. The effect of TPC2 on autophagosome accumulation could be attributed to an increase in lysosomal pH upon lysosomal Ca^2+^ release, which inhibits autophagy at the level of autophagosome–lysosome fusion (Figure 1). Therefore, lysosomal acidification could suppress TPC2-induced autophagosome accumulation (93). Similar findings of TPC2 have been observed in astrocytes, where its overexpression resulted in an increase in Beclin 1 and LC3-II levels (94). The latter may also relate to the accumulation of autophagosomes.

### Ca^2+^ Influx from Extracellular Environment

Recently, oxidative stress has been implicated in autophagy inhibition through induction of melastatin-related transient receptor potential cation channel member 2 (TRPM2)-mediated Ca^2+^ influx (95). Ca^2+^ influx resulted in the activation of Ca^2+^/calmodulin-dependent protein kinase II (CaMKII), which phosphorylated Beclin 1 at Ser295 and abolished its autophagy-inducing properties. The mechanism involved a decrease in Vps34 complex formation with phospho-Beclin 1 and increased Bcl-2 binding of phospho-Beclin 1 (Figure 1). Consequently to autophagy inhibition, oxidative stress triggered cell death in cells expressing TRPM2, while TRPM2 KD resulted in upregulated autophagy as a survival pathway in these cells. In addition, TRPM2/CaMKII activation further increased ROS production and contributed to mitochondrial fragmentation and loss of mitochondrial potential (95, 96).

## IP_3_Rs and Autophagy in Cancer

### IP_3_Rs in Cancer

IP_3_Rs control different hallmarks of cancer (97, 98). In particular, IP_3_Rs impact cell death and survival by mediating Ca^2+^ release from the ER and subsequently affecting mitochondria-regulated processes, including bioenergetics and apoptosis (99). Moreover, several IP_3_R isoforms can have distinct functions, dependent not only on their functional properties but also on their subcellular localization (100). For instance, IP_3_R3 has been particularly associated with pro-apoptotic Ca^2+^ flux from ER into mitochondria due to its localization at ER–mitochondrial contact sites (101, 102). As such, depending on the isoform and localization of the IP_3_R that is modulated, opposite effects can occur. Enhanced basal IP_3_R activity outside the MAMs may cause an increased passive Ca^2+^ leak from the ER. As a consequence, ER Ca^2+^ stores become less filled whereby less Ca^2+^ becomes available to be delivered to the mitochondria upon cellular exposure to a toxic, pro-apoptotic stimulus. By contrast, enhanced IP_3_R activity at the MAMs can increase not only mitochondrial bioenergetics but also the likelihood for pro-apoptotic Ca^2+^ transfers. In addition, Ca^2+^ transfer into the mitochondria also participate in oncogene-induced and replicative senescence, a stable proliferation arrest accompanied with distinct features like increased apoptosis resistance and altered gene expression (103–105). Here, IP_3_R2 and MCU were implicated in the enhanced mitochondrial Ca^2+^ transfer and accumulation that resulted in cellular senescence due to a decline in mitochondrial membrane potential and an increased ROS production and senescence. Therefore, loss of *ITPR2*, the gene encoding IP_3_R2, or of *MCU* overcame the growth arrest and escape from senescence (105, 106).

IP_3_Rs emerged as functional targets of an increasing number of oncogenes and tumor suppressors, which dynamically control IP_3_R activity and thus Ca^2+^ flux from ER into mitochondria (97, 107). Several oncogenes can suppress pro-apoptotic Ca^2+^-release events mediated by IP_3_Rs and this can occur *via* different mechanisms. First, oncogenes can directly interact with IP_3_Rs (97). For instance, anti-apoptotic Bcl-2 targets the central, modulatory domain of the IP_3_Rs, thereby suppressing excessive IP_3_R activity and protecting cells against pro-apoptotic Ca^2+^-release events (108–111). Alternatively, oncogenes can exert post-translational modifications of IP_3_Rs. Protein kinase B (PKB/Akt) phosphorylates IP_3_R3 and dampens its Ca^2+^ flux, suppressing pro-apoptotic Ca^2+^ transfer (112). Oncogenes not only prevent excessive IP_3_R-mediated Ca^2+^ release, but they can also promote basal Ca^2+^-signaling events that are associated with increased mitochondrial bioenergetics and, thus, increased NADH and ATP output. As such, Bcl-XL, another anti-apoptotic Bcl-2-family member sensitizes all IP_3_R isoforms, thereby promoting the occurrence of pro-survival Ca^2+^ oscillations and thus sustaining cell survival by boosting the mitochondrial metabolism (113–115).

Not only oncogenes but also tumor suppressors regulate IP_3_Rs (97, 107). The product of the BRCA1 gene, frequently mutated in breast cancer, binds and promotes IP_3_R activity. This underlies adequate apoptosis sensitivity of cells expressing wild-type BRCA1, while oncogenic mutations fail to engage IP_3_Rs and, thus, promote apoptosis resistance (116). Tumor suppressors can also act *via* post-translational modification of IP_3_Rs. As such, phosphatase and tensin homolog (PTEN) not only counteracts PKB/Akt activity by reducing phosphatidylinositol 3,4,5-trisphosphate (PIP_3_) levels but also reverses the PKB/Akt-dependent phosphorylation of IP_3_Rs, particularly at the MAMs where IP_3_R3 becomes dephosphorylated and de-repressed (117).

In addition to these events, oncogenic mutations in cancer genes can also affect IP_3_R expression levels. As such, the expression of mutant Ras in cells resulted in an IP_3_R-isoform switch, thereby reducing the pro-apoptotic IP_3_R3 isoform and increasing the pro-survival IP_3_R1 isoform (118). IP_3_R1 displays a higher IP_3_ sensitivity than IP_3_R3 and it is proposed to be less involved in pro-apoptotic Ca^2+^ transfers from ER into mitochondria than IP_3_R3. The elevated IP_3_R1-expression levels resulted in an increased Ca^2+^ leak from the ER and thus a slight decrease in ER Ca^2+^ content, which reduced Ca^2+^ availability for pro-apoptotic Ca^2+^ transfer at the ER–mitochondrial contact sites (118).

Finally, oncogenes and tumor suppressors can affect ER Ca^2+^ homeostasis by modulating other ER Ca^2+^-transport systems. For instance, the tumor suppressor p53 accumulates at ER membranes, targeting and boosting SERCA in cells exposed to toxic, chemotherapeutic, and photodynamic agents (119, 120). As a consequence, ER Ca^2+^ stores became overfilled and the likelihood to flood mitochondria with excess Ca^2+^ increased, underlying cell death induction by these agents. Cancer cells lacking p53 or having mutated p53 failed to increase SERCA activity and, thus, did not display increased mitochondrial Ca^2+^ overload, which contributed to the resistance of these cells to these toxic agents (119, 120). Recently, in neuroblastoma cells, acute application of cisplatin, a DNA alkylating agent, and topotecan, a topoisomerase I inhibitor, resulted in a rapid Ca^2+^ release from the ER stores (121). In addition to this, long-term exposure of neuroblastoma to these drugs resulted in a remodeling of Ca^2+^-transport systems, including an upregulation of IP_3_R and RyR isoforms. Blocking Ca^2+^ release from the ER by inhibiting these channels or by chelating cytosolic Ca^2+^ using a cell-permeable Ca^2+^ buffer, suppressed cell death in neuroblastoma treated with cisplatin and topotecan (121). For a detailed discussion on the impact of oncogenes and tumor suppressors on ER Ca^2+^ signaling and IP_3_R more specifically, we would like to refer to other recent reviews dedicated to this topic (107, 122).

In addition to this, altered IP_3_R expression has been implicated in a variety of cancer-associated processes. For instance, a subset of tumor tissue samples derived from breast cancer patients express higher IP_3_R2/IP_3_R3 levels compared to the adjacent non-tumorigenic tissue, which has been related to subsequent alterations in metabolic products (123, 124). IP_3_Rs were shown to be critical for the growth-stimulating effects of 17β-estradiol (E2) on tumorigenic MCF-7 breast cancer cells (125). E2 application exerted both acute and long-term effects on IP_3_Rs and Ca^2+^ signaling in MCF-7 cells. Acute E2 application triggered IP_3_R-mediated Ca^2+^ release, while prolonged E2 application resulted in an upregulation of IP_3_R3 expression. Further work indicated that IP_3_R3 and Ca^2+^-dependent K^+^ channels (BKCa) functioned in a concerted manner sustaining breast cancer cell proliferation by forming a macromolecular complex (126). Both IP_3_R3 and BKCa were critical for the proliferation of MCF-7 cells. Excitingly, in non-tumorigenic MCF-10A cells, IP_3_R3 and BKCa did not form such a complex and their expressions were dispensable for the proliferation of these cells (126). IP_3_Rs have also been implicated in the migration of cancer cells, a process associated by an epithelial–mesenchymal transition and stimulated upon loss of cell–cell contact (127). In disconnected pancreatic ductal adenocarcinoma (PDAC) cells, IP_3_Rs, together with Stim1-containing ER–PM junctions, redistributed to the leading edge of individual PDAC cells, supporting PDAC cell migration (127). Moreover, the selective inhibition of IP_3_Rs and SOCE lead to reduced cell migration underlying the importance of Ca^2+^ signaling in this process (127). Increased IP_3_R3 expression and IP_3_R-derived Ca^2+^ signals have also been shown to correlate with the invasive properties of glioblastoma cells (128, 129). Inhibition of IP_3_Rs with caffeine inhibited the invasion and migration of glioblastoma cells and increased the survival of mice xenografted with glioblastoma cells (128). Interestingly, not only inhibition of IP_3_Rs but also stimulation of IP_3_Rs suppresses glioblastoma cell growth and invasion. Indeed, trifluoperazine (TFP), a FDA-approved anti-psychotic drug, impeded proliferation, invasion, and motility of glioblastoma cells *in vitro* and *in vivo* by eliciting Ca^2+^ release from the ER through IP_3_R1 and IP_3_R2 channels, while IP_3_R3 channels were dispensable for TFP-induced Ca^2+^ mobilization. TFP-induced Ca^2+^ rise also depended on the presence of the calmodulin subtype 2 (CaM2) protein, which correlates with previous work revealing TFP as a calmodulin-inhibitory molecule by inducing a conformational change in Ca^2+^-calmodulin (130). Hence, it was proposed that TFP by targeting and antagonizing CaM2 alleviates CaM2’s inhibitory action on IP_3_Rs, resulting in a potent and irreversible Ca^2+^ release, responsible for the cell growth and invasion restraint of glioblastoma cells (129). More recently, IP_3_R-mediated Ca^2+^ signaling has been shown to be critical for normal T-cell development through repression of Sox1, an antagonist of the transcription factor Tcf1, which is important for normal T-cell development. In the absence of IP_3_R expression and activity, Notch signaling becomes active in T cells in post β-selection thymocytes, resulting in the development of aggressive T-cell acute lymphoblastic leukemia (131).

### Autophagy in Cancer

Autophagy is a basic catabolic process, existing in all types of cells, where it functions mostly in controlling protein turnover and sustaining energetic balance (132). In cancer, however, the role of autophagy is more complex and can exert different effects depending on the stage of tumor progression, tissue origin, genetic background, etc. Therefore, autophagy in cancer can serve as both a tumor suppressive and a tumor-promoting mechanism (133–135).

The essential autophagy protein Beclin 1, encoded by the gene *BECN1*, has been shown to act as a haploinsufficient tumor suppressor protein (136). In fact, mice that are haplo-deficient for *BECN1* develop spontaneous tumors due to an impaired basal autophagy and in humans, mutations in *BECN1* occur in up to 75% of breast, ovarian, and prostate cancers (137). Also other autophagy-involved proteins, such as UV radiation resistance-associated gene (UVRAG), Atg5, and Atg7, were recently described as tumor suppressors (137). These findings strongly support an oncosuppressive role of autophagy especially at early stages of tumor development. Autophagy contributes to the maintenance of cellular homeostasis, largely by degradation of protein aggregates and dysfunctional mitochondria but also by supplying nucleotides for DNA repair processes (138, 139). This further protects cells against proteotoxicity, oxidative stress, and genomic instability—the conditions supporting tumor development. In some settings, autophagy was also shown to be necessary to execute cell death to prevent tumor transformation (140). In p53-mediated cell death, expression of the lysosomal protein damage-regulated autophagy modulator-1 (DRAM-1), responsible for autophagy induction, was critical for apoptosis to occur (141). DRAM-1 was also shown to be downregulated in a subset of epithelial cancers (141) possibly underlying a similar tumor-suppressive function as Beclin 1.

In contrast to this, cancer cells become addicted to autophagy at the later stage of tumorigenesis. Studies from Guo et al. (142, 143) revealed that in *Kras-*driven, genetically engineered mouse models of non-small-cell lung cancer (NSCLC), deletion of *Atg7* caused accumulation of mitochondria, suppression of tumor growth and the promotion of tumor cell death. These data underscore the role of Atg7, which was required for NSCLC growth, survival, and malignancy. Furthermore, systemic genetic ablation of Atg7 in mice with established NSCLC, promoted tumor regression before damage occurred to the normal tissues (144). These findings indicate that tumors can be selectively autophagy dependent and that there exists a “therapeutic window” for autophagy modulation (144). In particular, autophagy could compensate metabolic stress by providing bioenergetics substrates for the TCA cycle and nucleotides for biosynthetic pathways, thereby supporting cancer cell survival (139). In *Kras*-driven NSCLC, autophagy-mediated recycling was able to sustain the levels of amino acids and several metabolites during starvation. However, further autophagy ablation caused deficiencies in mitochondrial substrates. Supplementation of glutamine, glutamate, and nucleotides was, therefore, critical to overcome autophagy deficiency caused by *Atg7* deletion which indicates the role of autophagy in starvation survival (139). Autophagy can also serve as a tumor pro-survival mechanism in response to chemotherapy and other anticancer treatments. It is known that the use of anticancer agents, such as 5-fluorouracyl, bortezomib, or tamoxifen, results in elevated autophagy, which counteracts cell death induction and decreases therapy efficiency (145). Recently, it has been described that verapamil, an L-type calcium channel blocker, can induce a cytotoxic effect and autophagy in colon cancer cells. Cell death in these cells was further increased when verapamil treatment was accompanied by chloroquine, an autophagy inhibitor, or when autophagy was ablated by *Atg5* and *Atg7* deletion (146). Usage of chloroquine and hydroxychloroquine is a common strategy for autophagy inhibition and, therefore, the enhancement of anticancer therapy effectiveness (147). Recent studies, however, have shown that chloroquine can exert anticancer effects independently of autophagy (148). In fact, chloroquine acted by reducing intratumoral hypoxia and metastasis, specifically normalizing tumor vessels by a mechanism involving NOTCH-1. Nevertheless, the use of autophagy inhibitors, as well as genetic tools to abort autophagy, strongly support the hypothesis of tumor-promoting effects of autophagy at the later stages of tumor development.

As discussed above, autophagy is most likely playing a tumor-suppressive role at early steps of tumor development, while it tends to function as a tumor-promoting mechanism in established tumors. This division, however, is not always clear as the role of autophagy in cancer can also depend on other factors such as the genetic background of a particular tumor. Recent studies revealed that in a humanized genetically modified mouse model of PDAC, deletion of autophagy genes *Atg5* and *Atg7* can play different roles according to the status of p53 (149). In mice possessing an oncogenic allele of *Kras*, ablation of *Atg5* and *Atg7* prevented further tumor development. However, in mice with mutated *Kras* and additionally lacking *p53*, blockage of autophagy significantly accelerated formation of tumor lesions (149, 150). This and other examples (151) indicate that the role of autophagy strongly depends on the tumor context and this should be considered while designing autophagy modulation-based therapies. For a further discussion on the role of autophagy in cancer, we refer to more detailed reviews (152–155).

## IP_3_Rs and Autophagy Control: A Role in Cancer?

### Autophagy Contribution to Modulation of Cancer Cell Death Induced by IP_3_R Inhibition

**Figure 2:**
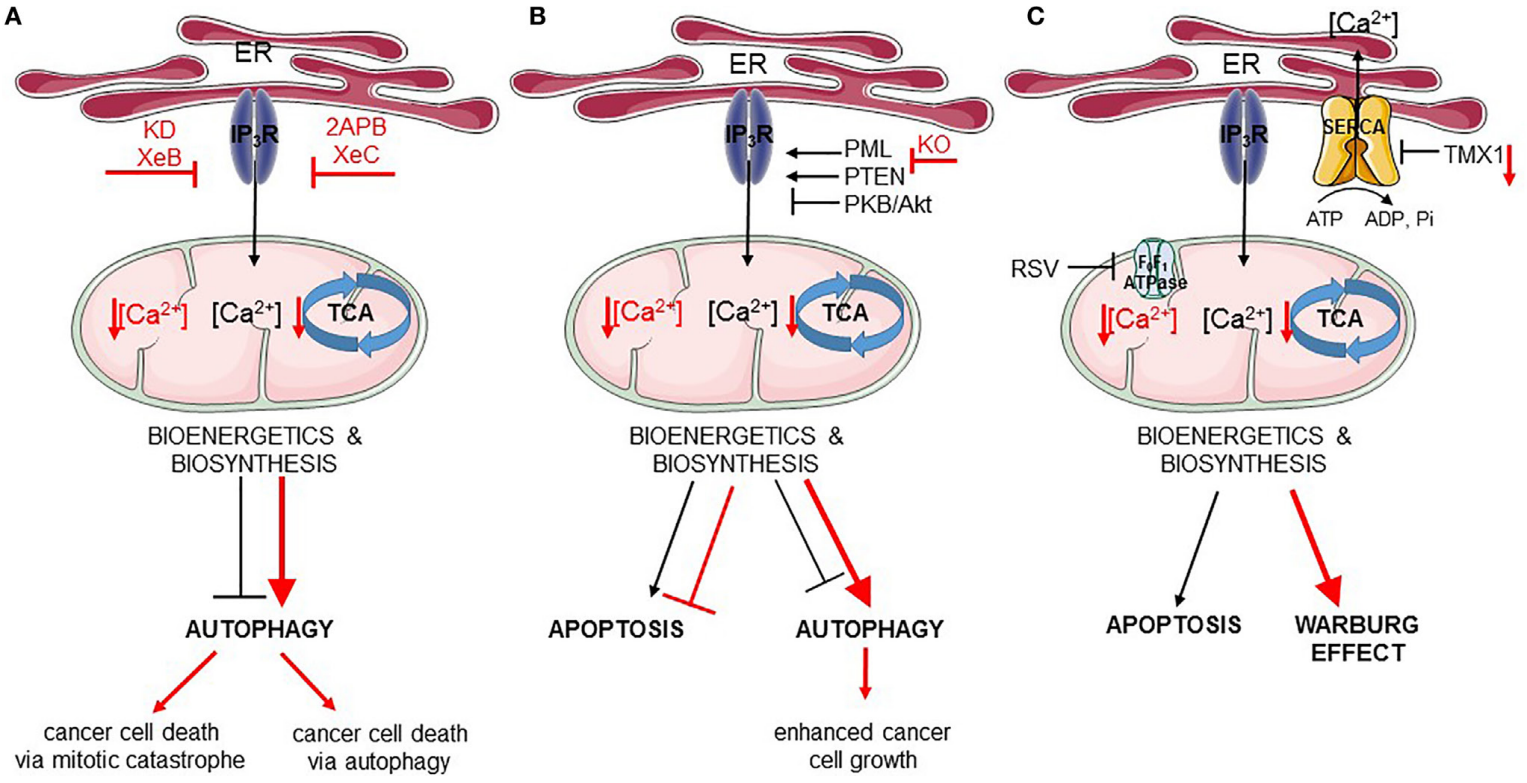
Endoplasmic reticulum (ER)–mitochondria Ca^2+^ transfer regulates apoptosis and autophagy in cancer. In all panels, the black arrows mirror the basal cellular mechanisms while the red arrows indicate modulation of the pathways by the listed chemical compounds or genetic modifications. **(A)** IP_3_Rs are engaged in autophagy and cell death regulation *via* the ER–mitochondrial Ca^2+^ flux. Ca^2+^ transferred to the mitochondria ensures proper tricarboxylic acid (TCA) function and, therefore, adequate bioenergetics and biosynthesis processes suppressing autophagy. Inhibition of IP_3_Rs by Xestospongin B (XeB) or its genetic knockdown (KD) dampens ER–mitochondrial Ca^2+^ transfer, which inhibits the TCA cycle and ATP production. As a consequence, autophagy is increased, but this is not sufficient for the survival of cancer cells, which undergo mitotic catastrophe. In addition to this, inhibition of IP_3_Rs by 2APB or Xestospongin C (XeC) also leads to impeded ER–mitochondrial Ca^2+^ fueling, and subsequently to further autophagy-dependent cancer cell death. **(B)** Fueling mitochondria with Ca^2+^ can be modulated by several oncogenes/tumor suppressors [promyelocytic leukemia protein (PML), PTEN, PKB/Akt]. In cancer cells lacking PML (KO, knockout), mitochondrial Ca^2+^ transfer is impeded resulting in downregulation of TCA cycle, stimulation of autophagy, and of cell growth. **(C)** Mitochondrial Ca^2+^ transfer can also be indirectly controlled by mitochondrial F_0_F_1_ ATPase. Its inhibition by resveratrol (RSV) impairs sarco/endoplasmic reticulum Ca^2+^ ATPases (SERCA) function, thereby increasing the net Ca^2+^ flux from the ER and promoting mitochondrial Ca^2+^ overload, eventually leading to apoptosis. In addition, SERCA is negatively regulated by thioreductase TMX1. Cancer cells having low levels of TMX1, exert high SERCA activity, which correlates with decreased ER–mitochondrial Ca^2+^ transfer. Consequently, the TCA cycle is limited and cells switch to aerobic glycolysis (Warburg effect).

Recently, it was shown that cancer cells may be addicted to basal IP_3_R activity and its critical role in feeding mitochondria with Ca^2+^, a regulator of the activity of several TCA cycle enzymes. Similarly to non-tumorigenic cells, in tumorigenic cells IP_3_R inhibition or KD resulted in an increased autophagic flux (156). However, while this was sufficient to sustain cell survival in non-tumorigenic cells, the increase in autophagy was not sufficient for the survival of cancer cells. Thus, pharmacological inhibition using xestospongin B or genetic KD of IP_3_Rs in cancer cells resulted in cancer cell death (156) (Figure 2A). Mitochondrial Ca^2+^ and its positive effect on the TCA cycle does not only serve to support ATP synthesis but also to support several anabolic pathways that use mitochondrial TCA cycle intermediates in their biosynthetic pathway (157). In non-tumorigenic cells, the lack of ER–mitochondrial Ca^2+^ transfers due to IP_3_R inhibition dampened cell cycle progression, thereby arresting cells at the G1/S checkpoint. Indeed, adequate ATP production is an integral part of the G1/S checkpoint and a surge in ATP production is needed for cells to progress from the G1 phase to the S phase *via* a mechanism that involves cyclin E upregulation (158). This surge in ATP output is achieved by mitochondrial hyperfusion at that stage of the cell cycle (159). In cells experiencing blunted ER–mitochondrial Ca^2+^ transfer (like upon IP_3_R inhibition), ATP output would be impaired and AMPK can be activated. One of the outcomes of AMPK activation besides autophagy induction is the activation of p53/p21, which downregulates cyclin E levels. As a consequence, cells cannot proceed from the G1 to S phase and are arrested (157). However, one feature of cancer cells is their uncontrolled proliferation. In fact, many cancer cells have mutations in p53 or display dysregulated cell cycle control (160). In that sense, these cancer cells become addicted to their mitochondrial metabolism to produce mitochondrial intermediates that are used for synthesis of lipids, nucleotides, and proteins, supporting the survival of the dividing cells. Cancer cells exposed to IP_3_R inhibitors will, thus, not slow down their cell cycling. As a consequence, these cells will continue to divide irrespective of the surge in mitochondrial ATP output and the availability of mitochondrial intermediates needed for biosynthesis upon cell division. Without sufficient lipids and nucleotides, the daughter cells will not be able to survive. Hence, IP_3_R inhibition caused a mitotic catastrophe in cancer cells. Consequently, cell death in these cells could be overcome by the addition of mitochondrial substrates, such as pyruvate, to the growth medium or by slowing down cell cycle progression (156). Yet, autophagy was not involved in the cell death process, as KD of essential autophagy genes did not modulate IP_3_R inhibition-induced cell death. This indicates that cell death did not occur *via* autophagic cell death and also that autophagy activation could not support survival of the cells.

In contrast to these findings, another study revealed an important role for autophagic cell death in breast cancer cells exposed to IP_3_R inhibition (123) (Figure 2A). Tumorigenic breast cancer cells were sensitive to treatment with the non-specific IP_3_R inhibitors, 2-APB or xestospongin C, while non-tumorigenic breast cancer cells were resistant to this treatment. However, these compounds, as well as their derivatives, can indirectly cause depletion of the ER Ca^2+^ stores by inhibiting other Ca^2+^ transporters or by stimulating Ca^2+^ leakage out of the ER (161–164). Nevertheless, similar findings were obtained by genetically knocking down IP_3_R2 or IP_3_R3, which appear to be upregulated in tumorigenic versus non-tumorigenic breast cancer cell lines. Inhibition of IP_3_Rs in the tumorigenic cells resulted in excessive autophagy activation, which could be attributed to a decrease in ATP production (and thus activation of AMPK), an upregulation of Atg5 and an increase in ROS production. In these cells, excessive autophagy was responsible for cell death, as inhibition of autophagy either at the level of the Vps34 complex formation using 3-methyladenine or at the level of lysosomal degradation by Bafilomycin A1 protected cells against cell death induced by IP_3_R inhibition or IP_3_R KD (123). These *in vitro* findings were also translated to *in vivo* xenografted breast tumor models. Interestingly, the increase in IP_3_R2/IP_3_R3 expression was not only found in breast cancer cell lines but also found in patient samples consisting of breast tumor tissue compared to adjacent non-tumorous tissue. Moreover, IP_3_R2/IP_3_R3 upregulation in breast tumors correlated with an increase in lipoproteins and several metabolites (such as lactate, alanine, and lysine) in the serum of these patients compared to breast cancer patients with low IP_3_R2/IP_3_R3 levels or healthy controls (124). Unfortunately, no autophagic markers were analyzed in these samples.

### Downregulation of Autophagy and Increased Apoptosis Susceptibility in Response to IP_3_R Modulation by Tumor Suppressors

As previously discussed, IP_3_Rs suppress basal autophagy by promoting mitochondrial ATP production. In addition to this, IP_3_Rs control the susceptibility of cells toward toxic, pro-apoptotic stimuli. By promoting Ca^2+^ transfer into the mitochondria, IP_3_Rs participate in mitochondrial Ca^2+^ overload, a critical factor in the opening of the mitochondrial permeability transition pore and subsequent apoptosis. In fact, mitochondrial Ca^2+^ overload has been shown to be a critical component of several pro-apoptotic stimuli, including chemotherapeutic drugs (165). These stimuli can trigger Ca^2+^ release from the ER. In addition, these compounds can trigger ER Ca^2+^ overload by activating SERCA in a p53-dependent manner (120). As a consequence, such cells will display an increased likelihood for mitochondrial Ca^2+^ accumulation and thus cell death. In fact, cancer cells lacking p53 or expressing loss-of-function p53 mutants are resistant to chemotherapeutic drugs in part due to their lack of mitochondrial Ca^2+^ overload, as these cells can be re-sensitized to chemotherapeutics by overexpressing MCU (120, 166). In particular, the IP_3_R3 isoform appears to participate in pro-apoptotic Ca^2+^ transfer into the mitochondria due to its presence in the MAMs (167). Moreover, IP_3_R3 activity in the MAMs is subjected to functional modulation by survival/oncogenes and tumor suppressors (Figure 2B). IP_3_R3 is phosphorylated by PKB/Akt, suppressing ER–mitochondrial Ca^2+^ flux and promoting apoptosis resistance (112). PKB/Akt-mediated phosphorylation of IP_3_R3 is counteracted by PTEN, which dephosphorylates IP_3_R3 particularly at the MAM fraction and, therefore, stimulates pro-apoptotic Ca^2+^ transfer from the ER into the mitochondria (117). Another regulator of PKB/Akt-dependent phosphorylation of IP_3_R3 is the tumor suppressor promyelocytic leukemia protein (PML), which also resides at the MAMs, where it recruits PP2A, which suppresses PKB/Akt activity (168). In addition to this, downregulation of IP_3_R3-protein levels has been implicated in cancer transformation and cell death resistance of isogenic cell pairs, in which an oncogenic mutant Ras allele was expressed (118).

More recently, further insights into the contribution of IP_3_R modulation in the tumor suppressive function of PML have been revealed (168, 169). PML was recruited at the MAMs in a p53-dependent manner supporting efficient ER–mitochondrial Ca^2+^ transfer (Figure 2B). As a consequence, cells expressing the tumor suppressor PML were susceptible to engage apoptosis upon cell stress or damage and to maintain an adequate production of ATP, preventing the growth of damaged or malignant cells. These conditions dampened AMPK activity and, thus, result in a regular basal autophagic flux. The combination of adequate apoptosis sensitivity and regular autophagy flux allows for a normal and balanced cell growth. However, in cells lacking PML, ER–mitochondrial Ca^2+^ transfer was suppressed, resulting in excessive apoptosis resistance and an upregulation of basal autophagy due to suppressed ATP production followed by AMPK activation and thus increased ULK1 activity. The increase in autophagy upon PML deletion in cells could be attributed to the decreased mitochondrial Ca^2+^ signaling, since overexpression of MCU in these cells could suppress basal autophagic flux (168). Moreover, PML-deficient cells displayed a growth advantage compared to PML-proficient cells, particularly in stress conditions such as nutrient starvation that engage the autophagy pathway. Interestingly, PML-deficient cells could be sensitized to chemotherapeutic drugs such as 5-fluorouracil by co-administration of chloroquine, an autophagy inhibitor that acts at the level of the lysosomal proteolysis. Moreover, in some promyelocytic leukemia cells, PML became fused to retinoic acid receptor α (RARα), abrogating wild-type endogenous PML function and causing neoplastic transformation. This oncogenic PML fusion was degraded by stimulating the proteasome using arsenic trioxide. Treatment of cells expressing oncogenic PML fusion protein with this drug not only resulted in PML-RARα degradation but also rescued wild-type PML levels, which was then present in the MAMs and able to promote ER–mitochondrial Ca^2+^ transfer (168).

Another tumor suppressor actively involved in autophagy regulation is Beclin 1. Interestingly, a target of Beclin 1 is the IP_3_R, which becomes sensitized upon Beclin 1 binding, a process enhanced during nutrient starvation (58). Thus, cancer cells, commonly deficient in Beclin 1, will not only be able to form less complexes with the lipid kinase Vps34 but also with the IP_3_Rs, what may lead to decreased autophagy. However, at this point, it is not clear how decreased IP_3_R/Beclin 1-complex formation contributes to basal autophagy and how this impacts tumorigenesis.

### Indirect Impact of IP_3_R on Autophagy in Cancer Cells: Effects of ER Ca^2+^ Modulation

Resveratrol, a natural polyphenol, is well known to induce cancer cell death by engaging autophagy induction *via* different mechanisms (82, 170–172), including the modulation of Ca^2+^ signaling (173). More recently, further insights into RSV-induced cancer cell death *via* Ca^2+^ signaling have been obtained (84). In particular, cancer cells can display increased ER–mitochondrial contact sites, potentially to facilitate the transfer of basal Ca^2+^ signals to accommodate their increased need for mitochondrial TCA cycle activity, which also provides substrates for several biosynthetic pathways. Exposing cancer cells to RSV resulted in a rapid depletion of the ER Ca^2+^ stores. The underlying mechanisms appeared to involve the direct inhibition of the mitochondrial F_0_F_1_-type ATP synthase, resulting in a rapid drop in ATP levels, in particular at the ER–mitochondrial contact sites (Figure 2C). SERCA activity is thereby impaired, leading to a net increase of Ca^2+^ delivery to the mitochondria, as for a given IP_3_-induced Ca^2+^ release less Ca^2+^ will be pumped back in the ER. Thus, mitochondrial Ca^2+^ levels will increase, thereby promoting cell death. The concept of SERCA modulating ER–mitochondrial Ca^2+^ transfer in cell death and tumor biology has been nicely illustrated in a recent study of Raturi et al. (174). It has been shown that SERCA activity was dynamically regulated at the ER–mitochondrial contact sites by different factors, including palmitoylated calnexin, which positively regulated SERCA, and the thioreductase TMX1, which negatively regulated SERCA. In normal cells, TMX1 levels are high, thereby suppressing SERCA activity and thus promoting ER–mitochondrial Ca^2+^ transfer. This supports mitochondrial metabolism on the one hand and adequate apoptotic susceptibility on the other hand. Interestingly, many tumors display low TMX1 levels, which results in increased SERCA activity, particularly at the MAMs, leading to dampened ER–mitochondrial Ca^2+^ transfer (Figure 2C). This was proposed to contribute to the Warburg effect and increased glycolysis, as the activity of TCA cycle enzymes became suppressed, while the need for glucose metabolism remained high to sustain cell growth and proliferation (174, 175). Nevertheless, it is possible that these findings relate to concepts identified for PML at the ER–mitochondrial interface. Indeed, tumors with low TMX1 levels may display increased autophagy and decreased apoptosis susceptibility due to suppressed ER–mitochondrial Ca^2+^ fluxes. However, further work is needed to reconcile these concepts.

### IP_3_R-Regulated Autophagy As a Protection against Natural Killer (NK)-Induced Cancer Cell Death

Recently, *ITPR1*, the gene encoding IP_3_R1, has been implicated as a major resistance mechanism of renal carcinoma cells against the lytic action of NK cells by activating autophagy (176–178). Many renal carcinoma cells are characterized by a dysfunctional von Hippel–Lindau gene (*pVHL*), which encodes a protein that has many functions, including targeting the family of hypoxia-inducible factor transcription factors for degradation by the proteasome. Thus, in cells lacking pVHL, HIF1α/HIF2α become stabilized. Currently, an emerging concept in anticancer therapies is the use of NK cells. Tumor cells contain several resistance factors against NK-induced cancer cell killing, including stabilized HIF2α. Strikingly, *ITPR1* appeared to be one of the most important target genes of HIF2α in a renal carcinoma cell line with dysfunctional *pVHL* gene and it conferred resistance against NK-induced lysis. In particular, renal carcinoma cells upregulated pro-survival autophagy in a HIF2α/IP_3_R1-dependent manner in response to NK treatment, while cells in which IP_3_R1 was knocked down failed to stimulate autophagy and became susceptible to NK-induced lysis. The IP_3_R1-dependent induction of autophagy protected the carcinoma cells against the deleterious action of NK cells by degrading the lytic granzyme B. In renal carcinoma cells with functional pVHL, HIF2α levels are very low due to its targeting to the proteasome, thus failing to upregulate *ITPR1* expression and abrogating autophagy induction as resistance mechanism against NK cells and the lytic action of granzyme B. Thus, antagonizing IP_3_R function in renal carcinoma cells lacking functional pVHL may provide a manner to sensitize these cells to lysis by NK cells by counteracting the induction of autophagy as a resistance mechanism. Furthermore, also NK cells by themselves require functional autophagy for maturation and survival (179). Upon deletion of *Atg5*, NK cells accumulated damaged mitochondria, which lead to their death due to excessive ROS production. Furthermore, silencing *Atg7* and the resulting disruption of the interaction between Atg7 and phosphorylated forkhead box O1 (FOXO1) prevented autophagy and contributed to incomplete maturation of NK cells (179).

## Conclusion

IP_3_Rs play a critical role in autophagy due to their localization at the ER and the ER–mitochondrial contact sites and the resulting Ca^2+^-signaling regulation. On the one hand, they suppress autophagy by continuously sustaining the mitochondria with Ca^2+^, needed for mitochondrial metabolism and energy production. On the other hand, they participate in the increased autophagic flux induced upon cellular stress, including nutrient starvation, chemical mTOR inhibition, or RSV treatment, by providing cytosolic Ca^2+^ that is needed to drive autophagic flux. Given autophagy’s critical role in tumor development and progression, it is not surprising that IP_3_R function can affect these processes through autophagy modulation. Cancer cells appear to be addicted to IP_3_R-mediated Ca^2+^ release to sustain their mitochondrial metabolism and related anabolic pathways. Cancer cells exposed to IP_3_R inhibition can undergo cell death, which in some cases could be due to excessive autophagy induction, while in other cases cell death could be the result of an uncontrolled cell proliferation and thus be due to mitotic catastrophe. Also, IP_3_Rs are modulated by tumor suppressors, such as PML, as part of a homeostatic program to support normal cell growth by balancing adequate apoptosis susceptibility and regulated autophagy flux. However, loss of these tumor suppressors results in dampened IP_3_R function and thus defective ER–mitochondrial Ca^2+^ transfer. As a consequence, cells become resistant to cell death inducers, including genotoxic stress and cell damage, by a combination of increased apoptosis resistance and an increased autophagy flux serving as a pro-survival function. This phenomenon will contribute to neoplastic transformation and tumorigenesis. The role of IP_3_Rs in autophagy seem also to be exploited by renal carcinoma cells with dysfunctional pVHL, which induce autophagy in a HIF2α/IP_3_R-dependent manner as a resistance mechanism that protects these cancers against NK-induced killing. Finally, it should be noted that IP_3_R function and the net IP_3_R-mediated Ca^2+^ delivery into the mitochondria is dependent on the activity of SERCA, which is also present in the MAMs. Thus, SERCA modulation at the ER–mitochondrial contact sites will affect the net Ca^2+^ transfer into the mitochondria and, thus, ultimately affect the Ca^2+^-dependent mitochondrial functions, such as bioenergetics, autophagy, and apoptosis. SERCA inhibition in the MAMs will increase mitochondrial Ca^2+^ accumulation, which will drive the mitochondrial metabolism and bioenergetic output and thus suppress autophagy. In cancer cells, de-inhibition of SERCA at the ER–mitochondrial contact sites can be part of the Warburg effect but also of the increase in basal autophagy that could promote neoplastic behavior by promoting cell survival and excessively protecting cancer cells against cell stress. Overall, IP_3_Rs impact several cancer hallmarks through autophagy modulation.

## Author Contributions

All authors contributed to the conception of the work. GB and EK drafted the manuscript. EK produced the figures. GR, TV, and JP critically revised manuscript and figures and provided important intellectual content. All authors concur with the final version of the manuscript and agree to be held accountable for all aspects of the work.

## Conflict of Interest Statement

The authors declare that the research was conducted in the absence of any commercial or financial relationships that could be construed as a potential conflict of interest.
